# Nasal nitric oxide in allergic rhinitis in children and its relationship to severity and treatment

**DOI:** 10.1186/s13223-017-0191-z

**Published:** 2017-04-04

**Authors:** Peng-peng Wang, Gui-xiang Wang, Wen-tong Ge, Li-xing Tang, Jie Zhang, Xin Ni

**Affiliations:** Department of Otorhinolaryngology Head and Neck Surgery, Beijing Children’s Hospital, Capital Medical University, 56 Nan Li Shi Road Xi Cheng District, Beijing, 100045 People’s Republic of China

**Keywords:** Allergic rhinitis, nNO, Quality of life, Nasal symptoms

## Abstract

**Background:**

Nasal nitrous oxide (nNO) is increased in allergic rhinitis (AR), but not in asthma, and is a non-invasive marker for inflammation in the nasal passages.

**Methods:**

Levels of nNO were measured and compared in healthy children and children with mild and moderate-to-severe AR. Levels of nNO before and after treatment with steroids and/or antihistamine were then compared in the 2 AR groups. Their relationship to quality of life and nasal symptom and reactivity to outdoor and outdoor allergens were examined.

**Results:**

nNO levels were higher in mild AR than in healthy children and higher in moderate-to-severe AR than in mild AR. One month steroid and/or antihistamine treatment lowered nNO levels to control levels in mild AR and approximately halfway to control levels in moderate-to-severe AR. nNO levels had a weak correlation to quality of life questions and a fair correlation to nasal symptom scores before treatment. This correlation was weakened or lost after treatment, and no correlation was seen between nNO levels and responses to indoor or outdoor allergens.

**Conclusion:**

nNO levels in children with AR may be useful for assessing the response to treatment. Their relationship to quality of life, nasal symptoms, and sensitivity to specific allergens needs further study.

## Background

Nitrous oxide (NO) is produced by eosinophils and airway epithelial cells in response to inflammation [1]. Fractional exhaled NO (FeNO) from the lower airways, although increased in allergic rhinitis (AR), is increased to a greater extent in asthma and has been used in diagnosis and to follow treatment of this condition [2–7].

Nasal nitrous oxide (nNO) is produced in the nasal cavity and sinuses. More NO in expired air is from the nasal cavity than the lower airways [1]. A number of studies have shown that nNO is decreased in conditions in which the sinus becomes blocked (nasal polyps adenoidal hypertrophy), ciliary function is compromised (ciliary dyskinesia) or inducible NO synthase in the airway epithelium is reduced (cystic fibrosis) [8–12]. A few studies have shown nNO to be increased in AR [4, 10, 13] and one study has reported that anti-histamine treatment decreased the elevated nNO levels seen in AR patients [14].

nNO is a non-invasive marker of inflammation that is increased in AR, and can easily be measured during a routine office visit [9, 10]. We wished to see whether nNO measurement might be useful clinically in determining AR severity and following the patient’s response to treatment. Therefore, in the current study we measured nNO in healthy children, children with mild AR, and children with moderate-to-severe AR before and after a 1 month treatment with steroid with or without antihistamine. We then determined the relationship between nNO concentrations and AR severity, treatment, quality of life, nasal symptomatology, and response to specific allergens.

## Methods

### Patients

This prospective study enrolled children aged 3–14 who had been diagnosed with allergic rhinitis and healthy, non-allergic children of the same age group. After informed consent had been obtained from the patient’s legal guardians, children were eligible to be recruited into present study.

The study was approved by the Institutional Review Board of Beijing Children’s Hospital, Capital Medical University (2015-133). Informed consent was obtained from the study participants, and the consent was written.

#### Allergic rhinitis group

The patients were defined as those who had been diagnosed with allergic rhinitis (AR) according to the allergic rhinitis and its impact on asthma (ARIA) guidelines [15]. Inclusion criteria were (1) patients had one or more of the following toms: sneezing, watery nasal discharge, nasal congestion and nasal itching (eye symptoms such as itching and conjunctival hyperemia might also be present), (2) the skin prick test (Allergopharma@, NHD; Merck, Germany) had been performed for 16 standard allergens and was positive (++ = 75%, +++ = 100%, +++ = 125% of the patient’s reaction to histamine, which was about 3 mm in diameter) or detection of serum sIgE with 20 standard Euroline Chinese allergens was positive (EUROIMMUN, Germany), (3) symptoms were present for ≥4 days per week for a consecutive 4 weeks.

Exclusion criteria were (1) concomitant upper or lower airway inflammatory disease such as nasal polyps, sinusitis, or asthma, (2) abnormal nasal anatomical structure or previous nasal surgery, (3) abnormal heart and/or lung function, (4) respiratory infection during the previous week, (5) smoking, (6) a history of systemic or local treatment with glucocorticoids.

The 2008 ARIA guidelines [15] were used to classify type and severity of rhinitis All subjects in the rhinitis group had persistent allergic rhinitis (defined as symptoms present ≥4 days per week for a consecutive 4 weeks), rather than intermittent allergic rhinitis.

#### Normal control group

Healthy children who received routine physical examination were recruited from the Health Care Center for the normal control group. They were children 3–14 years old in whom AR had been excluded after reviewing the medical history and physical examination. Children with other allergic diseases, or with immunodeficiency diseases, diabetes, tuberculosis or asthma were excluded.

Patients with a history of upper and lower respiratory tract surgery were excluded from both groups.

#### Treatment

According to the severity of symptoms and the impact of AR on quality of life (such as sleep, daily life, working and learning), AR patients were further divided into two subgroups: mild AR and moderate-to-severe AR. Mild severity was no troublesome symptoms or impairment of function and moderate/severe severity was one or more of the following: sleep disturbance; impairment of daily activities, leisure and/or sport; impairment of school or work; troublesome symptoms [15]. And on the basis of the recommendations in the ARIA guideline, clinicians asked the guardians to medicate the patients with mometasone furoate alone or mometasone furoate combined with antihistamine. Treatment lasted for 1 month and then re-examination was performed.

### Data collection

#### Baseline characteristics

Results of allergy testing, age, gender, height, body weight, and treatment were recorded.

#### Subjective evaluation of nasal symptoms

Before and after the treatment period, AR-related symptoms were scored by the patients and their guardians. Scoring was done according on the intensity of the following symptoms: rhinitis, sneezing, runny nose, nasal congestion, nasal itching. A visual analog scale (VAS) was used to score the symptoms.

#### Scoring of quality of life

The Rhinoconjunctivitis Quality of Life Questionnaire *(*RQLQ) for children aged 6–12 years was used for the evaluation of quality of life both before and after the treatment period. This questionnaire uses a 6 point scale to record the responses to 24 questions about the effect of AR on quality of life.

#### Detection of nasal nitric oxide

Nasal nitric oxide (nNO) was measured with the nanocoulomb breathalyzer (Sunvou Medical Electronics) according to the criteria developed by American Thoracic Society (ATS) and European Respiratory Society (ERS) in 2005 [16] and the Product Registration Guideline of FDA of USA in 2003. The unit of nNO is ppb (parts per billion) (1 ppb = 1 × 10^−9^ mol/L). For nNO detection, one nostril is occluded with a latex nasal olive, and a tube attached to the olive that leads to the detector Air is withdrawn from the nostril by a suction pump at a standard speed through a central sampling channel in the olive. Pumping is done for 10 s while the subject, after a deep inhalation, exhales against a resistance provided by an oral roll placed in his/her mouth to occlude exhalation through the mouth. The un-occluded nostril allows escape of the nNO exhaled from the lung. The instrument displays the measured nNO concentration. When the measurement has been completed, the olive is removed, placed in the other nostril, and the detection process repeated. The mean of the NO values for the two nostrils is used as the final value.

Patients were asked to eat no food during the 3 h before the examination. In the detection process, inhalation of air containing NO > 10 ppb was avoided by inhalation of air through an NO filter. Intense exercise or examinations such as those for detection of lung function were avoided during the hour previous to the examination. During the examination, air leakage, taking a breath, breath-holding and spitting were avoided. The examinations were all performed by the same clinician to order to avoid inter-observer differences.

### Statistical analysis

Data on age, body mass index (BMI), and age at diagnosis, are presented as mean ± standard deviation (SD). Other continuous parameters are shown as median and interquartile range (the range between P_25_ and P_75_). Gender distribution is expressed by count (%) and tested by Chi square test. To examine differences in continuous variables between the control group and the two subgroups of AR patients, one-way analysis of variance (ANOVA) and the Kruskal–Wallis test were performed. When a significant difference was revealed by the Kruskal–Wallis test, the Mann–Whitney U test was then performed for post hoc tests. The differences between patients with mild and moderate to severe rhinitis were examined by independent sample *t* test or Mann–Whitney U test. The difference in change in nNO between AR patients and controls was tested by the Mann–Whitney U test as well. The Wilcoxon signed rank test was carried out to examine the post-treatment change in nNO concentration for each group. Correlations between nNO concentrations and RQLQ questionnaire scores or VAS scores of five nasal symptoms were quantified by Spearman’s rank correlation. According to the correlation coefficient, 5 levels of correlation were defined: very weak (<0.2), weak (0.2–0.4), fair (0.4–0.6), strong (0.6–0.8), very strong (≥0.8). All statistical assessments were evaluated at a two-sided alpha level of 0.05 using IBM SPSS software, version 22 (IBM Corp., Armonk, NY, USA).

## Results

Table 1 summarizes differences between controls and the two groups of AR patients. Age, gender distribution, and BMI were similar in the three groups, but pre-treatment nNO concentrations were significantly different. Those with moderate-to-severe AR had significantly higher nNO than those with mild AR and controls, and those with mild AR had significantly higher nNO than controls.

**Table 1:** Characteristics of children with no allergic rhinitis (AR), mild AR, and moderate-to-severe AR

	Control (n = 49)	Mild (n = 50)	Moderate+ (n = 44)	*P*
Age, years	7.2 ± 1.9	6.1 ± 2.3	6.8 ± 2.3	0.053
Sex				0.135
Female	27 (55.1)	32 (64)	33 (75)	
Male	22 (44.9)	18 (36)	11 (25)	
BMI, kg/m^2^	17 ± 3.5	16.9 ± 4.5	16.8 ± 3	0.955
Pre-treatment nNO (ppb)	220.5 (186.5, 240.5)	279.5 (257, 323)^a^	497.5 (447.5, 635.5)^a,b^	*<0.001*

Data are shown as mean ± standard deviation for age and BMI, median (interquartile range) for pre-treatment nNO, and frequency (%) for gender

Italic value indicates significant difference among the three groups, *P* < 0.05

^a^Indicates significant difference from the control group, *P* < 0.017

^b^Indicates significant difference from mild AR group, *P* < 0.017

**Fig. 1:**
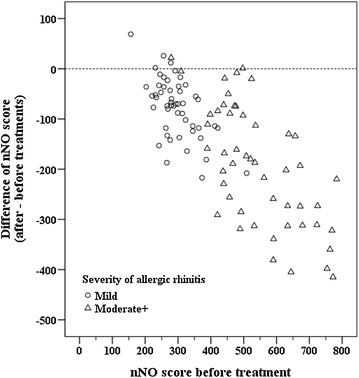
Scatter plot of change in nNO score after treatment vs. pre-treatment score

**Fig. 2:**
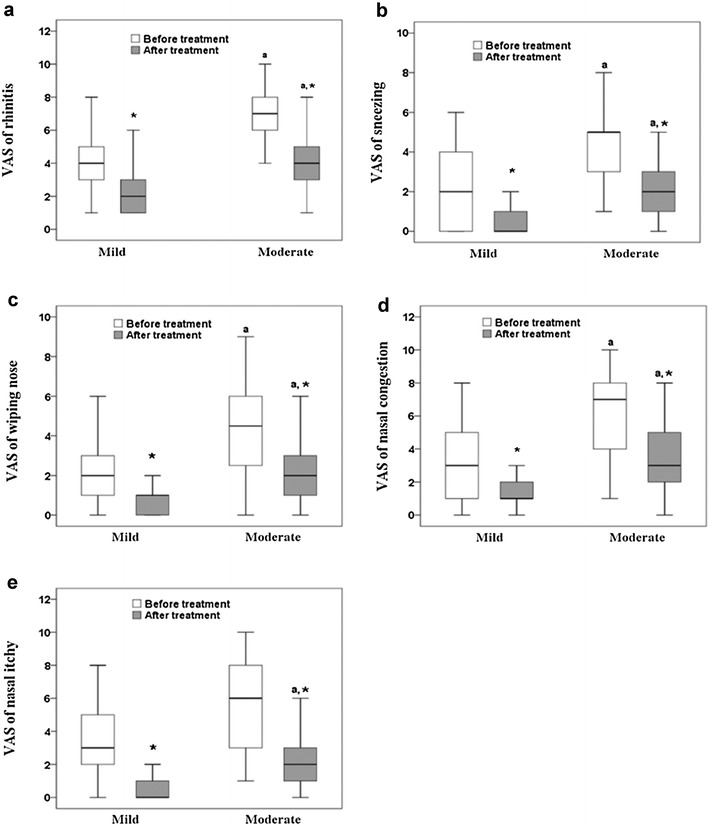
Pre- and post-treatment visual analog scale (VAS) scores of **a** rhinitis, **b** sneezing, **c** runny nose, **d** nasal congestion, and **e** nasal itchiness. *Box plot* consists of median (middle line), the P_25_ (*bottom* of the box) and the P_75_ (*top* of the box) percentiles. Mann–Whitney U test and Wilcoxon signed rank test are implemented to examine group difference at two time points and post-treatment changes of VAS scores for each group, respectively. Letter *a* denotes significant difference from mild group. *Asterisk* denotes significant change after treatment

Differences between mild and moderate-to-severe groups are further analyzed and summarized in Table 2. Individual decreases in nNO score after treatment compared to pre-treatment values are shown in Fig. 1. Patients with mild AR were diagnosed at a later age than those with moderate-to-severe AR (4.5 vs. 4.2 years, *P* < 0.001), and the duration of the AR was shorter in the mild AR group (12 vs. 24 months, *P* < 0.001). Quality of life scores, as expected, were significantly worse in the moderate-to-severe group than in the mild group. Both AR groups showed a decrease in nNO after treatment, and this decrease was significantly higher in the moderate-to-severe group than in the mild group (*P* < 0.001). Similar results were found in changes of VAS scores of all allergic symptoms (*P* < 0.001) (Fig. 2).

**Table 2:** Differences between children with mild or moderate-to-severe allergic rhinitis

	Mild (n = 50)	Moderate+ (n = 44)	*P*
Age at diagnosis, years	4.5 ± 2.1	4.2 ± 2.1	*<0.001*
Disease duration, months	12 (4, 24)	24 (8, 48)	*<0.001*
Post-treatment change in nNO concentration, ppb	−69.5 (−118.0, −36.0)	−191.0 (−301.0, −90.0)	*<0.001*
RQLO, points^a^	0.17 (0, 0.96)	2.08 (1.02, 2.64)	*<0.001*
Inconvenience due to stuffy nose	0 (0, 2)	4 (0, 4.5)	*<0.001*
Inconvenience due to sneezing	0 (0, 1)	2 (1, 3)	*<0.001*
Inconvenience due to runny nose	0 (0, 1)	2 (0.5, 3)	*<0.001*
Inconvenience due to itchy nose	0 (0, 2)	2 (0.5, 4.5)	*<0.001*
Inconvenience due to itchy eyes	0 (0, 1)	2 (0, 3.5)	*<0.001*
Inconvenience due to watery eyes	0 (0, 0)	1 (0, 1.5)	*0.001*
Inconvenience due to red eyes	0 (0, 0)	1 (0, 2)	*0.001*
Inconvenience due to swollen eyes	0 (0, 0)	0.5 (0, 1)	*<0.001*
Inconvenience due to painful eyes	0 (0, 0)	0 (0, 1)	*<0.001*
Rubbing nose or eyes repeatedly	0 (0, 2)	3 (1, 5)	*<0.001*
Postnasal drip	0 (0, 2)	3 (1, 5)	*<0.001*
Having to carry handkerchiefs	0 (0, 0)	2 (0, 4)	*<0.001*
Having to take medications	0 (0, 0)	0 (0, 1)	*<0.001*
Interference with leisure activities	0 (0, 0)	1 (0, 3)	*<0.001*
Inconvenience due to dry throat	0 (0, 0)	1 (0, 2.5)	*<0.001*
Inconvenience due to itchy throat	0 (0, 0)	2 (0, 3)	*<0.001*
Inconvenience due to headache	0 (0, 0)	0 (0, 1)	0.054
Inconvenience due to fatigue	0 (0, 1)	1.5 (0, 3)	*<0.001*
Interference with mood	0 (0, 0)	2 (0, 3.5)	*<0.001*
Interference with attention	0 (0, 1)	2 (0, 4)	*<0.001*
Interference with sleep	0 (0, 1)	1 (0, 2.5)	*<0.001*
Frequent nocturnal awakening	0 (0, 1)	1 (0, 2)	*0.001*
Irritable/anxiety/angry	0 (0, 1)	2 (0, 4)	*<0.001*
Embarrassed because of nasal symptoms	0 (0, 0)	1 (0, 1)	*<0.001*

Age at diagnosis is presented as mean ± standard deviation and tested by independent sample t-test; the others are shown as median (interquartile range) and tested by Mann–Whitney U test

Italic value indicates significant difference among two groups, *P* < 0.05

*nNO* nasal nitric oxide, *RQLQ* Rhinoconjunctivitis Quality of Life Questionnaire

^a^Scoring system of RQLQ questionnaire: *0* no effect, *1* hardly any effect, *2* mild effect, *3* moderate effect, *4* medium effect, *5* major effect, *6* severe effect

The relationship of nNO concentrations before and after treatment to quality of life scores, and VAS scores for allergic symptoms is summarized in Table 3. Before treatment, nNO concentrations were positively and significantly, although weakly, related to 23 of the 24 items in the RQLQ (*P* ≤ 0.042). After treatment, only 7 of the 24 items in the RQLQ retained this weak, although significant relationship to nNO concentration (*P* ≤ 0.037). Before treatment, weak or fair correlations with nNO concentrations were seen for all VAS scores for allergic symptoms (all *P* < 0.001). These correlations weakened but retained significance after treatment.

**Table 3:** Relationship of nNO concentration to RQLQ quality of life scores and VAS scores for five nasal symptoms after treatment for allergic rhinitis

	Pretreatment nNO concentration		Post-treatment nNO concentration	
	*γ*_*s*_	*P*	*γ*_*s*_	*P*
RQLQ, points	0.402	*<0.001*	0.521	*<0.001*
Inconvenience due to stuffy nose	0.300	*0.003*	0.140	0.179
Inconvenience due to sneezing	0.372	*<0.001*	0.168	0.106
Inconvenience due to runny nose	0.328	*0.001*	0.112	0.281
Inconvenience due to itchy nose	0.287	*0.005*	0.120	0.249
Inconvenience due to itchy eyes	0.339	*0.001*	0.091	0.383
Inconvenience due to watery eyes	0.276	*0.007*	0.230	*0.026*
Inconvenience due to red eyes	0.211	*0.042*	0.084	0.419
Inconvenience due to swollen eyes	0.283	*0.006*	0.181	0.081
Inconvenience due to painful eyes	0.255	*0.013*	0.265	*0.010*
Rubbing nose or eyes repeatedly	0.334	*0.001*	0.136	0.190
Postnasal drip	0.345	*0.001*	0.181	0.081
Having to carry handkerchiefs	0.349	*0.001*	0.189	0.068
Having to take medications	0.212	*0.041*	0.133	0.200
Interfere with leisure activities	0.327	*0.001*	0.277	*0.007*
Inconvenience due to dry throat	0.349	*0.001*	0.292	*0.004*
Inconvenience due to itchy throat	0.304	*0.003*	0.224	*0.030*
Inconvenience due to headache	0.158	0.127	0.162	0.120
Inconvenience due to fatigue	0.311	*0.002*	0.117	0.260
Interference with mood	0.366	*<0.001*	0.183	0.077
Interference with attention	0.371	*<0.001*	0.210	0.042
Interference with sleep	0.279	*0.006*	0.062	0.552
Frequent nocturnal awakening	0.257	*0.012*	0.070	0.502
Irritable/anxiety/angry	0.359	*<0.001*	0.220	*0.033*
Embarrassed because of nasal symptoms	0.388	*<0.001*	0.215	*0.037*
VAS score, point				
Allergic rhinitis	0.540	*<0.001*	0.389	*<0.001*
Sneezing	0.441	*<0.001*	0.373	*<0.001*
Runny nose	0.408	*<0.001*	0.296	*0.004*
Nasal obstruction	0.477	*<0.001*	0.288	*0.005*
Nasal itchiness	0.361	*<0.001*	0.283	*0.006*

Spearman’s rank correlation is implemented, and italic value indicates a significant association with concentration of nNO, *P* < 0.05

*γ*_*s*_ correlation coefficient of Spearman’s rank correlation, *NO* nitric oxide, *RQLQ* Rhinoconjunctivitis Quality of Life Questionnaire

Responsiveness to indoor or outdoor allergens showed no relationship to post-treatment decreases in nNO concentrations (all *P* > 0.05) (Table 4). However, more post-treatment change in nNO concentration was noted as the VAS score for the symptom of runny nose increased, although the other VAS scores for nasal symptoms were not related to the decrease in nNO seen after treatment.

**Table 4:** Relationship of post-treatment change in nNO concentration to the allergy testing results and to post-treatment changes in VAS scores for symptoms

Allergy testing	Change in nNO concentration	
	Median (interquartile range)	*P* ^a^
H6 test		
Indoor allergens		0.932
No	−102 (−204, −52)	
Yes	−93 (−187, −54)	
Outdoor allergens		0.403
No	−89 (−180, −50)	
Yes	−133 (−217, −70)	
Skin prick test		0.883
Indoor allergens		
No	−91 (−202, −47)	
Yes	−111 (−193, −55)	
Outdoor allergens		0.904
No	−93 (−193, −52)	
Yes	−113 (−204, −47)	

Changes in visual analog scale (VAS) score	Correlation coefficient	*P* ^b^
Overall VAS	0.123	0.237
Sneezing	0.023	0.823
Runny nose	0.300	*0.003*
Nasal obstruction	0.181	0.081
Nasal itchiness	0.174	0.093

Mann–Whitney U test^a^ and Spearman’s rank correlation^b^ are implemented

Italic value indicates statistical association with change in amount of NO, *P* < 0.05

## Discussion

In the current study of pediatric patients with AR, increased nNO was related to increased disease severity, increased VAS scores for nasal symptoms, and decreased RQLQ quality of life scores. Treatment with steroids and/or antihistamines caused a greater nNO decrease in moderate-to-severe AR than in mild AR and weakened the relationship between nNO and quality of life and nasal symptom scores. The magnitude of the post-treatment decrease in nNO bore no relationship to sensitivity to indoor or outdoor allergens.

The drugs and doses used for treatment of AR in this study were selected according to clinical judgement, not to a set treatment plan. Therefore we do not have the data to determine to what degree the greater post-treatment drop in nNO in the more severe AR group was due to more aggressive drug treatment in this group. However, although it seems paradoxical at first glance, the smaller drop in nNO in the mild AR group represented greater clinical success than the larger drop seen in the moderate-to-severe group. This is because of the very high pre-treatment nNO concentrations seen in the moderate-to-severe group. The 70 ppb drop in nNO concentrations in the mild group brought ppb concentrations down to normal levels, while the almost 200 ppb drop in concentrations in the moderate-to-severe group brought the ppb concentrations only halfway down to normal.

Treatment weakened the correlation between nNO and quality of life and VAS nasal symptom scores. For RQLQ quality of life scores, the correlation was weak before treatment and was lost for most of the items after treatment. For the VAS nasal symptoms scores, the correlation was stronger than for quality of life scores, fair correlation before treatment in most cases, and decreased to weak after treatment. The stronger correlation of nNO to nasal symptom than quality of life scores is probably because symptoms were easier to assess accurately. For example, the “sneezing” score in the nasal symptom list should be easier to assess accurately than the corresponding “inconvenience due to sneezing” in the quality of life list.

Several possible reasons exist for the post-treatment decrease in correlation between nNO and nasal symptoms. After treatment, when the inflammation has been controlled, the patient may still be troubled by symptoms. That is, the nNO level may be perfectly correlated to the inflammatory status, but fail to correlate to the nasal symptoms until the nNO level has stabilized for a period. In addition, we only determined the nNO level, and the continuing discomfort of some patients might be due to undetermined inflammation elsewhere. Or after treatment, the degree of discomfort may be too low to be evaluated accurately, a condition that would make the correlations disappear after treatment.

It is difficult to compare nNO concentrations in this study to those of other studies, because although nNO levels in AR have been measured in other studies, the patients are usually adults or are a cohort in which patients with both AR and asthma are included. Liu et al. do report nNO levels in children with AR but no asthma, but do not distinguish between mild and moderate-to-severe AR, so the results are not comparable to ours [13]. The single study of the effect of treatment on nNO in AR [14 **]** that a 2 week treatment with the antihistamine levocetrizine decreases nNO from 800 to 700 ppb in adults and adolescents who were actively experiencing symptoms. Quality of life was improved, but the authors did not attempt to correlate individual quality of life scores to nNO concentrations, as was done in our study.

A limitation of the study was that we were unable to perform a correlation between the overall VAS score and nNO score because we had no valid combination rule to use to perform this comparison. The lack of correlation between nNO levels and the results of allergy testing may be because too many other factors affect allergy test results. It would be useful in the future to compare nNO levels in allergic and non-allergic rhinitis to see whether determination of nNO levels could be substituted for skin prick testing in distinguishing between the two groups.

## Conclusions

In conclusion, our results suggest that nNO measurements can be used clinically for the pre-and post-treatment evaluation, but use of nNO for evaluation of severity or to find the specific allergens that cause the AR needs more study.

## Abbreviations

AR: allergic rhinitis
FeNO: fractional exhaled NO
nNO: nasal nitrous oxide
NO: nitrous oxide
